# Retraction: metastatic collecting duct carcinoma of the kidney treated with Sunitinib

**DOI:** 10.1186/1477-7819-9-136

**Published:** 2011-10-24

**Authors:** El Mehdi Tazi, Ismail Essadi, Mohamed F Tazi, Youness Ahellal, Hind M'rabti, Hassan Errihani

**Affiliations:** 1 Department of Medical Oncology, National Institute of Oncology, Rabat, Morocco; 2 Department of Urology, CHU Hassan II, Fez, Morocco

## Retraction

The authors have retracted this article [1] as it contains large portions of text and figures that have been duplicated from another article previously published in the *International Journal of Clinical Oncology *[2]. Lead author Dr Tazi accepts full responsibly for the duplication and would like to apologise to the co-authors, Editors and readers as well as the authors of the original article.
